# Comparison of Adaptive Spectral Estimation for Vehicle Speed Measurement with Radar Sensors

**DOI:** 10.3390/s17040751

**Published:** 2017-04-02

**Authors:** Khairul Khaizi Mohd Shariff, Edward Hoare, Liam Daniel, Michail Antoniou, Mikhail Cherniakov

**Affiliations:** Microwave Integrated Systems Laboratory, University of Birmingham, Birmingham B15 2TT, UK; KXM330@bham.ac.uk (K.K.M.S.); e.g.hoare@bham.ac.uk (E.H.); l.y.daniel@bham.ac.uk (L.D.); m.antoniou@bham.ac.uk (M.A.)

**Keywords:** speed over ground, Doppler radar sensor, vehicle speedometer, speed estimation algorithm

## Abstract

Vehicle speed-over-ground (SoG) radar offers significant advantages over conventional speed measurement systems. Radar sensors enable contactless speed measurement, which is free from wheel slip. One of the key issues in SoG radar is the development of the Doppler shift estimation algorithm. In this paper, we compared two algorithms to estimate a mean Doppler frequency accurately. The first is the center-of-mass algorithm, which based on spectrum center-of-mass estimation with a bandwidth-limiting technique. The second is the cross-correlation algorithm, which is based on a cross-correlation technique by cross-correlating Doppler spectrum with a theoretical Gaussian curve. Analysis shows that both algorithms are computationally efficient and suitable for real-time SoG systems. Our extensive simulated and experimental results show both methods achieved low estimation error between 0.5% and 1.5% for flat road conditions. In terms of reliability, the cross-correlation method shows good performance under low Signal-to-Noise Ratio (SNR) while the center-of-mass method failed in this condition.

## 1. Introduction

The availability of a precise speed measurement system is vital for an effective implementation of safety systems such as anti-braking system (ABS) and traction control system (TCS) in automobiles. At present, wheel encoder is widely used as a speed sensor that counts the wheel rotation to estimate the vehicle speed [1]. This system is well known to provide reliable estimate of speed when driving on a high-friction surface. However, when driving on low-friction surfaces, such as wet, uneven or steep surfaces, the speed estimate can have a considerable amount of error due to wheel slip. For this reason, automakers require accurate speed sensors for optimal operation of vehicle safety system.

In the literature, several contactless sensors have been proposed to estimate vehicle speed. These include Lidar (Laser), ultrasonic, acoustic and Global Positioning (GPS) satellites [2,3,4,5]. Nevertheless, it is also known that the performance of these sensors is limited in practice. Laser undergoes heavy attenuation in rain, fog and snow conditions [6]. Ultrasonic sensors are susceptible to noise and cross-talk [7]. In [3], the authors show that an acoustic sensor is not reliable when vehicle moving at high speed. The GPS system can provide accurate speed, but is unavailable in underground roads. For these reasons, alternative types of sensors operating on different physical principles are desirable. Radar is an alternative sensor which is robust against extreme environment such bad weather conditions and high temperature. In addition, owing to advancements in sensor technology, radar sensors are relatively inexpensive and physically small enough to be fitted on a vehicle. Due to these reasons, radar has been recognized as a promising sensor for true vehicle speed measurement [8,9,10,11,12].

Radar can be classified according to its architecture. Generally radar is divided into three types: continuous wave radar (CW); frequency-modulated continuous wave radar (FMCW); and pulse radar [13]. The latter is not attractive for speed measurement due to its broadband nature. Short distance measurement requires radar to generate very short pulses with high bandwidth. Consequently it needs an expensive, high-speed analog-to-digital converter (ADC) to capture the signal [14]. In contrast, FMCW and CW radar work by radiating continuous transmission of waves. FMCW radar provides Doppler and range information of a moving object and CW radar only provides Doppler information. These radars consume less power than pulse radar during its operation, which make them favorable for automobile applications. Although both FMCW and CW radar can measure speed, CW radar is preferred in the application of vehicle speed estimation due to its low cost and simple signal processing [15,16].

Doppler shift estimation is a critical task in SoG radar systems. Robust and accurate estimation methods remains a challenge. In addition, apart from high-accuracy requirements, an automotive radar system should be low-cost due to car mass production, and provide information as quickly as possible so that this information can lead to timely action from the ground vehicle’s other systems or its driver. These factors point to the use of low-cost, typically CW sensors, and computationally efficient processors with the goal of measuring Doppler frequency and converting it to a SoG estimate. 

Techniques such as the zero-crossing method have been proven to deliver high-accuracy speed estimation [17,18]. However, is known to be unreliable on wet and icy roads [19]. The problems involved in the estimation stems from the frequency spread caused by the limited beam width of the radar’s antenna, as well as the amplitude fluctuations near the mean frequency of the Doppler spectrum caused by the individual random scatterers in the illuminated region of the radar [20]. In addition, the random existence of high-amplitude spurious peaks and low-frequency noise (1/f noise) can easy prevent the algorithm from finding the correct Doppler shift.

The noisy nature of the Doppler signal has motivated the development of several Doppler shift algorithms in the frequency domain. One of the simplest methods is to use location of the center-of-mass of spectrum as the Doppler shift frequency. This method assumes that the spread of power across a spectrum follows a Gaussian distribution, thus the location of the peak can be calculated using a simple average method. An extended version of this algorithm can be found in [21], where the algorithm excludes frequencies with no significant amplitude in computing the Doppler shift, in order to reduce the algorithm bias.

Another approach is to use spectrum-template matching. This method measures similarity between the measured spectrum and a known shape determined by prior knowledge of the spectrum creation. A method proposed by [19,22] finds the Doppler shift by correlating multiple pre-determined spectra with the measured spectrum. In this formulation, the algorithm selects the index of the best-matching template, and has been shown to be of sufficient accuracy. However, this technique is computationally intensive as it involves multiple correlations, and is therefore time-consuming, which is a restriction in automotive environments, particularly in emerging automotive areas such as autonomous ground vehicles.

This paper introduces two new techniques for SoG estimation, designed for automotive applications. These methods rely on low-cost radar sensors (wide-beam, continuous wave) to estimate speed-based measured Doppler spectra analysis, and additionally taking into account the overall computational efficiency. However, both algorithms have their own relative merits and drawbacks, so their performance in realistic road conditions over extensive measurements should be assessed. It should be noted here that to the authors’ knowledge there are no commercial radars in production for this purpose in cars yet, so only a relative performance between the two algorithms is possible. Therefore, the purpose of this paper is two-fold: it aims to (a) introduce the two new algorithms for SoG estimation; and (b) compare their relative performance for different road environments through a dedicated experimental campaign. This paper is organized as follows: Section 2 presents an overview of the SoG measurement principle and describes the nature of the Doppler spectrum. Section 3 proposes algorithms, including the execution steps, in detail. Section 4 presents their bias and variance in the presence of white noise in the spectrum. Section 5 describes the performance of the algorithms tested with Doppler signal collected from actual road surfaces. Finally, Section 6 contains the summary of this work.

## 2. SoG Fundamentals

### 2.1. Speed Measurement Principle

**Figure 1:**
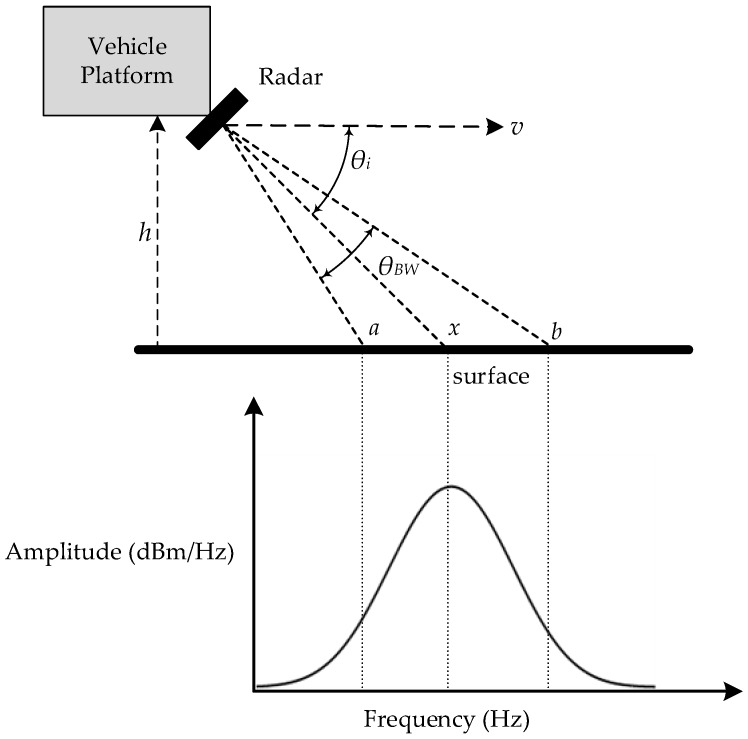
A typical speed-over-ground (SoG) radar setup on vehicle and the resultant Doppler Spectrum.

A Doppler sensor measures vehicle speed by radiating continuous waves to the ground. The waves are then scattered by the ground and the frequency is changed by an amount proportional to the vehicle speed. A Doppler shift caused by one scatterer on the ground is given by the classic Doppler formula

(1) $$f_{d}=\frac{2v}{\lambda }\cos\theta_{i}$$

where *v* is the vehicle speed above the ground, *λ* is the wavelength of the transmitted signal, *θ_i_* is the inclination angle of the sensor with respect to ground plane and *f_d_* is the Doppler shift frequency. Figure 1 illustrates a geometrical SoG setup and the resultant Doppler spectrum due to the antenna beam width. In this setup, a radar sensor with antenna beam width *θ_BW_* is mounted on a car at height *h*, and looking at the ground at angle *θ_i_* with respect to the direction of travel. For simplicity, we consider Figure 1 as a two-dimensional setup, which means that the antenna only has a beam width in the direction of travel and no beam width (beam width = 0°) in the transverse direction.

The radar sensor illuminates a strip of the ground bounded by angle *θ_BW_*. The resultant echo signal forms a spread of frequency where the amplitude reaches maximum when the radar is directly illuminates the point *x*. The large majority of the echo energy is confined around the echo center frequency and the power far away from the center frequency is negligible. The 3-dB beam width spread is approximately equal to [21]

(2) $$\Delta f_{d}=\frac{2v}{\lambda }\left[\cos\left(\theta_{i}-\frac{\theta_{BW}}{2}\right)-\cos\left(\theta_{i}+\frac{\theta_{BW}}{2}\right)\right]$$

(3) $$\Delta f_{d}\approx \frac{2v}{\lambda }\theta_{BW}\sin\theta_{i}$$

where Δ*f_d_* is the Doppler spread. This demonstrates that any practical radar with finite antenna beam width will generate a spread of frequency of Δ*f_d_*. For example, a SoG radar system with antenna beam width *θ_BW_* = 15°, antenna look angle *θ_i_* = 45°, transmitter frequency of *f_T_* = 24 GHz and with vehicle travelling at *v* = 10 ms^−1^ produces a relative Doppler spread Δ*f_d_*/*f_d_* of 1.3. To achieve speed estimation error of 0.5%, Δ*f_d_*/*f_d_* must be in order of 0.1. This shows that the transmitter antenna is required to have very narrow beam. In practice, small antenna beam width requires physically large antenna size, which is not suitable for automobiles application. On the other hand, signal-processing techniques can improve the estimation accuracy.

### 2.2. The Doppler Spectrum

**Figure 2:**
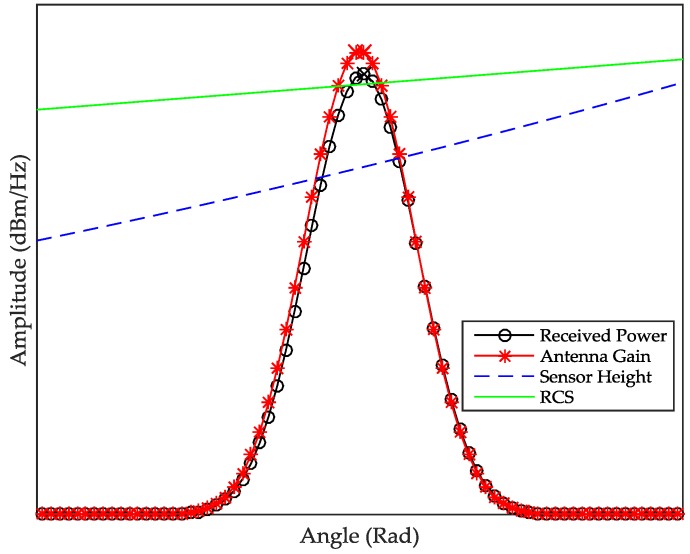
The comparison between radar return power and its parameters.

Again, consider the SoG setup in Figure 1. The radar receiver antenna collects much backscattering power from discrete points on the ground between points *a* and *b* with each point providing their own level of energy and Doppler frequency. The shape of spectral distribution along the radar bore sight can be determined using the radar total return power equation [13]

(4) $$P_{r}(\theta )=k\frac{\sigma (\theta )G^{2}(\theta_{i}-\theta )\sin^{4}(\theta )}{h^{4}}$$

(5) $$\theta =\cos^{-1}\left(\frac{f_{d}\lambda }{2v}\right)$$

where *θ* is the angle of the antenna radiation pattern with (*θ* = 0°) from the antenna boresight, *σ* is radar cross section (RCS) of the ground, *G* is the antenna gain, *h* is the height of the radar above the ground, *k* is the constant of the system and *P_r_* is the incident power density. For modeling simplicity, we assumed that the antenna gain has a Gaussian pattern in its first 3-dB power and the ground surface is uniform. Figure 2 shows the modeled Doppler spectrum.

Equation (4) shows that the shape of the Doppler spectrum depends only on *σ*, RCS and *G*. Specifically, the shape around the peak energy of the spectrum is largely determined by the antenna gain pattern. Note that there exists a slight shift in the Doppler peak position (marked with a cross) compared to the antenna pattern. This bias is due to RCS and sensor height. In practice, the whole shape of the spectrum may show statistical characteristics of a Rayleigh distribution [22]. This is because the gain under the 3-dB beam width is sufficiently strong, producing a Doppler curve with a long tail on the far side on the antenna beam width. Furthermore, the Doppler curve is not smooth but spiky because the vehicle moves above the road where each scatterer radiates a signal slightly different in amplitude and phase compared to the sensor transmitter.

## 3. Proposed Adaptive Mean Frequency Estimation Methods

This section discusses all the necessary points required for the mean frequency estimation task. To begin, Section 3.1 explains the pre-processing which is necessary to remove unwanted noise generated from imperfections in the experimental instruments. Next, Section 3.2 describes the center-of-mass algorithm (CMA) and the pre-processing needed to achieve robust speed estimation. Similarly, Section 3.3 describes the cross-correlation algorithm (XCA).

### 3.1. Pre-Processing

In order to follow the requirements of the ABS [23], which requires speed to be updated in every 0.1 s, the time-series Doppler signal is framed into small fixed-length samples, *x*(*n*) of duration of less than 100 ms. We assumed the sample in the short frame is statistically stationary and the mean value for the Doppler signal should be equal to zero. Thus, subtracting the mean value from the signal will remove the DC signal in the samples. Next, to reduce distortion, we balanced the amplitude level between the radar’s in-phase and quadrature signal by measuring the gain between the two signals. The DC removal and gain balancing are given by the below equations

(6) $$x\prime (n)=x(n)-\frac{1}{n}\sum_{i}^{n}x(i)$$

(7) $$Gn=\frac{Var(I)}{Var(Q)}$$

where *x*’(*n*) is the sample with no DC offset, *Gn* is the amplitude gain and *I* and *Q* are the In-phase and quadrature Doppler signal. Finally, we transform *x*’(*n*) to a frequency data, *f*(*n*) using the Fast Fourier Transform (FFT) algorithm.

### 3.2. Center-of-Mass Algorithm (CMA)

Mathematically, the mean Doppler frequency is related to the first moment of the power spectrum and is defined as [24]

(8) $$f_{0}=\frac{\int_{f_{\min}}^{f_{\max}}fS(f)df}{\int_{f_{\min}}^{f_{\max}}S(f)df}$$

where *S*(*f*) is the RMS spectrum of the Doppler signal. *f*_0_, *f_min_* and *f_max_* are the mean, minimum and maximum frequency of the Nyquist sampling bandwidth of an ADC. Quite often sampling is done at a factor of 4–5 times larger than the required bandwidth of maximum vehicle speed. Integrating the mass across the bandwidth increases the bias and variance of the first moment of computation in Equation (8), thus it is desirable to have a pre-processing scheme that can minimize the integration limits of the narrow bandwidth of the Doppler signal. The problem is estimating the new minimum *f_min_*’ and maximum *f_max_*’ limits in the presence of random peaks and low-frequency noise. Finding *f_min_*’ and *f_max_*’ in these conditions may result in obscure results. Therefore, our goal is to design a robust pre-processing technique, purposely to mitigate the weight of these noises in the identification of the useful bandwidth. In this work, we divide the spectrum into small fixed-width windows and measure the energy content of each window. This reduces the effects of random peaks in determining the location of *f_min_*’ and *f_max_*’. The details of the algorithm are described as follows

Step 1. Firstly, measure the RMS spectrum via the FFT algorithm.
Step 2. The algorithm searches for the returned Doppler signal in the frequency domain. To do this, the algorithm uses an amplitude threshold that turns all signals above it to one and all signals below it to zero.
  (9) $$D(n)=\left\{ \begin{array}{l} 1,\;S(f)>TH_{1}\\ 0,\;S(f)<TH_{1} \end{array}\right.$$
  where *TH*_1_ is the system threshold. Naturally, the Doppler signal is corrupted by noise, so a suitable *TH*_1_ must be chosen relative to the noise floor. This of course implies that the SNR is sufficiently high, but given the relatively short distance between the ground and the sensor this assumption can be made. In our work, *TH*_1_ is given by
  (10) $$TH_{1}=\mu_{nf}+3\sigma_{nf}$$
  where *μ_nf_* and *σ_nf_* are the mean and standard deviation of the spectrum noise floor. We assumed our noise floor is normally distributed.
Step 3. Finding *f_min_*’: The algorithm searches *f_min_*’ from *f* = 1 toward the end of the spectrum by finding trailing of ones larger than a pre-determined width, *w.* Here, it is important to set a proper value of *w* so that the algorithm can distinguish between a true a tail of Doppler signal or a tail of random spurious peak. A noise burst can be ignored by the algorithm if value of *w* is larger than the width of a typical random spurious peak. However, a large value of *w* may not work well with narrow Doppler signals near the DC signal. Therefore, in this work we have set two different values of *w* for two different ranges of Doppler shift frequency.
  (11) $$w=\left\{ \begin{array}{ll} 5,\; & f_{d\;}<1000\;\mathrm{Hz}\\ 10,\; & f_{d}>\;1000\text{​ }\mathrm{Hz} \end{array}\right.$$
  where *f_d_* is the Doppler shift frequency and it is obtained by measuring the strongest frequency component from the smoothed version of the Doppler spectrum, *f*(*n*). A rough estimate of *f_d_* is used to select a value of w and it does not introduce deficiency in the final estimate of Doppler shift frequency.
Step 4. Finding *f_max_*’: The process of determining *f_max_*’ is similar in steps 3 except, the search starts from the end to the first sequence of the spectrum bins.
Step 5. The mean Doppler frequency is calculated by integrating the mass between *f_min_*’ and *f_max_*’ and the frequency location, where the mass balanced on both sides is taken as the Doppler shift frequency.
  (12) $$\sum_{f_{n}=f_{\min}\prime }^{f_{0}}f(n)=\sum_{f_{n}=f_{0}}^{f_{\max}\prime }f(n)$$
Step 6. Finally, vehicle speed is calculated using Equation (1).

### 3.3. Cross-Correlation Algorithm (XCA)

Cross-correlation is a measure of similarity of two signals as a function of sample-lag to one of the two signals. Cross-correlation is computed by shifting the reference signal across the length of the return echo and integrating the inner product on each sample across the spectrum length [25]. In practice, it is sufficient to perform a correlation equal or less than half the width of the sampling frequency. In the case of the CW Doppler radar, the return echo is a version of the reference signal with amplitude corrupted with noise and shifted by amount of *f* sample. One of the weaknesses of this technique is the unavailability of the reference signal. In SoG radar, the exact shape of the return echo is not known. Several ways of generating a reference signal is by using theoretical antenna pattern [26] or by collecting reference templates from experiments [27]. However, in practice, even with these references is available, the actual return echo may experience shape distortion in terms of amplitude and phase shift with respect to the reference pattern, due to surface conditions and performance of the radar system over time. In addition, the limited number of FFT samples for processing the return echo produces highly variable amplitude variation around the mean of the Doppler spectrum.

In this work, we proposed the use of cross-correlation between the measured Doppler spectrum and a Gaussian curve to estimate the Doppler shift. We assume that the Doppler spectrum has a Gaussian characteristic in the first of its half-power. This assumption is based on the theoretical model from Equation (4) and it is experimentally observed. A Gaussian curve is defined as

(13) $$P(f)=A_{f}\exp\left[\frac{{\left(f-f_{i}\right)}^{2}}{2\sigma^{2}}\right]+B_{f}$$

where *A_f_*_,_ *f_i_*, *σ* and *B_f_* are the parameters of the Gaussian curve. To establish a Gaussian curve using information from a Doppler spectrum, we assumed as follows: *f_i_* is the strongest frequency component of the smoothed Doppler spectrum with amplitude of *A_f_*, 2*σ* is the 3-dB beam width of the antenna pattern, which is approximately equal to Δ*f_d_* and *B_f_* is the amplitude shift. In details, the algorithm operates as follows:

Step 1. The spectral data is calculated via FFT algorithm.
Step 2. Next, the spectrum is smoothed using a moving averaged filter. This helps to reduce the effects of random peaks in the spectrum.
Step 3. At this stage, one frequency with the largest amplitude assumed to be the mean Doppler frequency. This allows us to grossly estimate the parameter *v* and consequently the σ of the Gaussian curve.
  (14) $$v=\frac{f_{a}\lambda }{2\cos\theta_{i}}$$
  (15) $$\sigma \approx \frac{v}{\lambda }\theta_{BW}\sin\theta_{i}$$
  where *f_a_* is the frequency component with the largest amplitude. This step does not introduce accuracy deficiency in the final speed estimation.
Step 4. The generated curve is correlated with the Doppler spectrum and the position with the highest correlation is assumed as the mean Doppler frequency. A correlation window can be used to limit the correlation length between the two signals to reduce the algorithm computation time.
Step 5. Finally, vehicle speed is calculated using Equation (1).

### 3.4. Evaluation of Bias and Standard Deviation Using Simulated Signal

**Figure 3:**
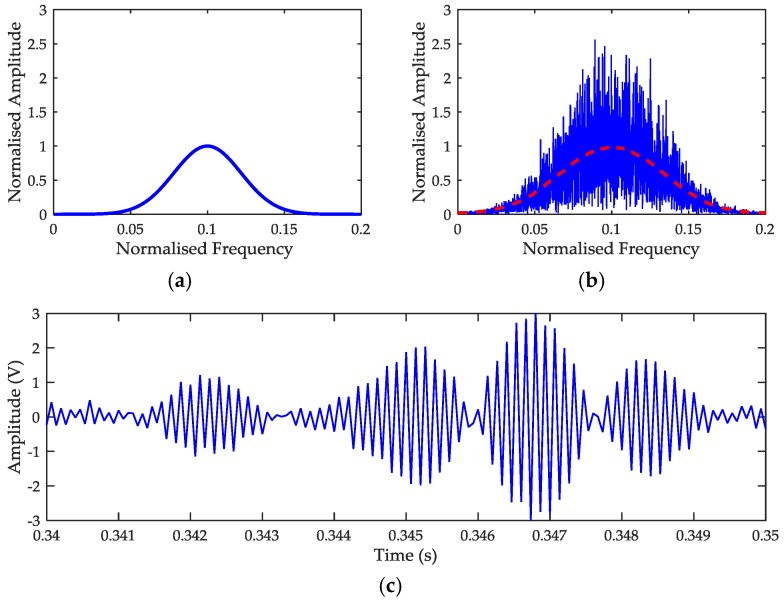
Simulated (**a**) Noise-free Doppler spectrum; (**b**) Doppler spectrum + noise; (**c**) Time-domain signal of the echo in Figure 3b.

We examine the bias and standard deviation produced by the algorithms using simulated Gaussian echoes. A return echo from a radar sensor with transmitter frequency *f_c_* = 24 GHz, antenna beam width *θ_BW_* = 15° and antenna look angle *θ_i_* = 45° is generated when mean frequency *f*_0_ = [100 200 … 2000] Hz and SNR = [0 10 … 50] dB. A zero-mean white Gaussian noise with variance *σ*^2^ is added to the echoes to forms a statistical amplitude variation similar to the spectrum collected from an actual Doppler sensor. The simulated signals are shown in Figure 3. To replicate the actual processing of SoG radar system, we transformed the echoes into time-series signals. These echoes are sampled at sampling frequency *f_s_* = 25 KHz, and transformed to spectral data using FFT with 2048 samples. Finally, we applied the Center-of-Mass Algorithm (CMA) and Cross-correlation Algorithm (XCA) to the simulated signal to estimate the *f*_0_. 

**Figure 4:**
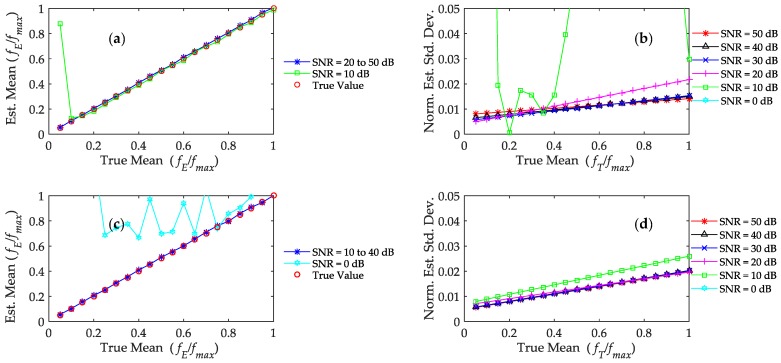
The bias and standard deviation produced by the algorithms (**a**) Center-of-Mass Algorithm (CMA) bias; (**b**) CMA standard deviation; (**c**) Cross-correlation Algorithm (XCA) bias (**d**) XCA standard deviation. The results for SNR = 0 dB is not presented (except in 4c) in the figures because the estimated mean and standard deviation at SNR = 0 dB is way above the scale of the figures.

Figure 4a,c show the mean bias for 1000 estimates of *f*_0_ for both algorithms. The red dots in the graph indicate the points where the true frequency is equal to the estimated frequency. By comparing these two plots, it can be seen that both methods achieve low estimation bias for SNR between 20 and 50 dB. However, at SNR = 10 dB, the two algorithms yield different results where CMA produces inaccurate measurement for *f*_0_ less than 200 Hz. In contrast, the XCA method works fine at this SNR level. Both algorithms fail at the lowest SNR = 0 dB.

Figure 4b,d present the standard deviation of the estimated *f*_0_ across different SNR. From both graphs, it can be observed that as SNR degrades, the mean frequency estimates deviate further than their true values. In a similar manner, we also observed the increase in deviation as the mean Doppler frequency increases, which is attributed to the broadening of the Doppler spectrum. The estimation using CMA produces large and inconsistent deviation at SNR = 10 dB. On the other hand, the estimation using XCA shows little increase in deviation. Both algorithms produce inconsistent deviation at SNR = 0 dB.

### 3.5. Computational Complexity

Both algorithms have linear complexity, O(*N_FFT_*) to number of FFT samples. To show the actual time required to complete an estimate of speed, measurements were performed using a computer with 3.6 GHz microprocessor and 16 GB of memory. We ran the algorithm using MATLAB and we estimate the time required to complete one estimate of speed using the MATLAB *tic* and *toc* operands. The actual Doppler data was sampled at 25,000 samples per seconds. Table 1 presents the mean computational times for several FFT lengths.

It is observed that both algorithms achieved small processing time compared to the duration of the samples, which is required for a fast update rate implementation in a SoG system. Comparatively, the CMA is faster than the XCA method by at least 30 times.

## 4. Experimental Data Collection

### 4.1. Data Acquisition System

**Figure 5:**
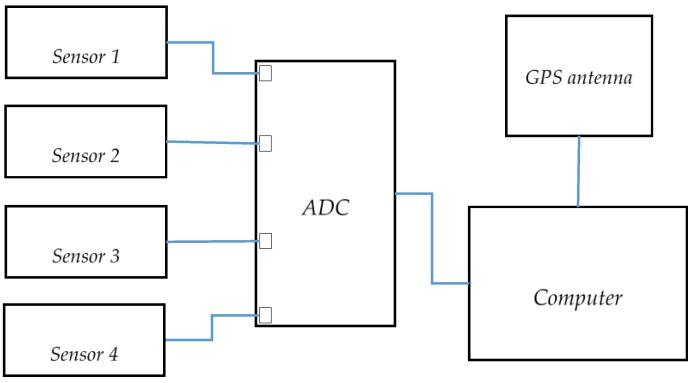
Measurement setup.

The speed of the test vehicle was measured simultaneously by 4 Doppler radars and a GPS receiver during a series of runs under varying speeds and different surface conditions. Figure 5 illustrates the block diagram of the radar system used in the experiment. In our setup, we used Doppler sensor module, K-MC1 produced by RF Beam GmbH. This sensor is a K-band transceiver operating at 24 GHz. The sensor is an all-in-one module which comprises of an RF front-end and an IF mixer and amplifier. The antenna has 25°/12° beam width in the horizontal and vertical direction respectively. It produces two Doppler outputs—in-phase and quadrature—allowing the detection of direction of the vehicle relative to the sensor. An ADC samples the Doppler signals at 15 KHz per channel and a computer is used to store the Doppler signal for further processing.

A low-cost GPS receiver consisting of a SiRF chipset was mounted on roof of the vehicle to provide full view of sky at all times. The receiver is connected to a computer via USB connection and programmed to produce National Marine Electronics Association (NMEA) output at maximum rate of 1 Hz. The NMEA data contains speed above ground data and several other navigation parameters. For this experiment, only the speed data is used.

### 4.2. Janus Configuration and Test Vehicle

**Figure 6:**
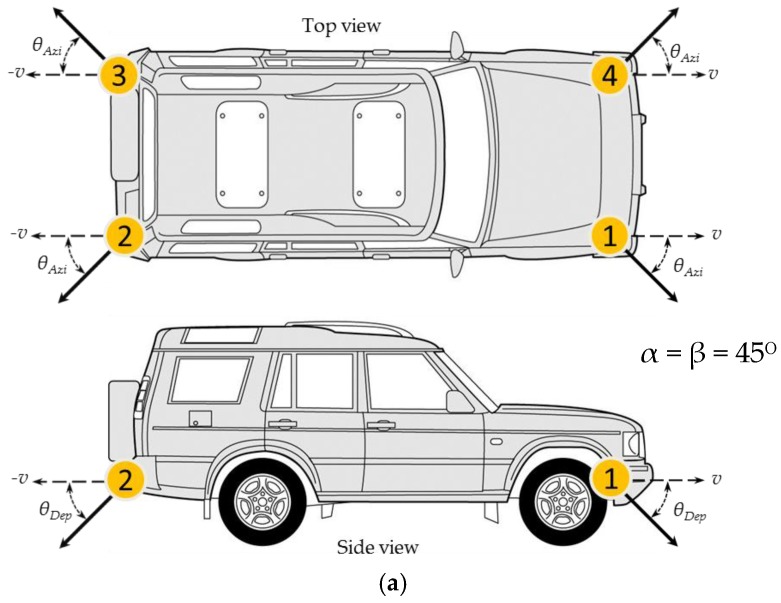

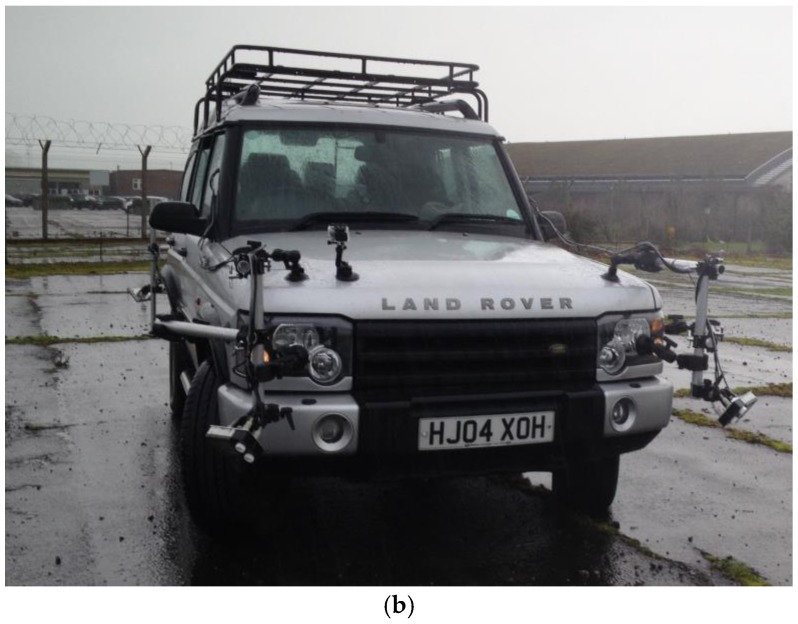
(**a**) A 4-radar Janus configuration. Top and side view of the test vehicle showing the depression and azimuth angle of the radar sensors; (**b**) A 4-radar Janus configuration. An actual image of the test vehicle with radars and a video camera installed.

We implemented a four-beam Janus configuration to reduce the effects of the vehicle pitch and roll in the speed computation. A sensor is mounted at each corner of the vehicle with the antenna facing downward 45° from the longitudinal speed direction, then 45° from the lateral speed direction. All sensors are mounted 0.5 m from the ground. The vehicle longitudinal speed is measured from the four sensors. Figure 6a shows the physical arrangement of sensors on the test vehicle. The test vehicle used in the experiment was a Land Rover Discovery with 3.0-L engine as shown in Figure 6b. Prior to test, we ensured all four tires have equal amount of pressure. A video camera was also installed to record the ground image during the experiment.

### 4.3. Experimental Site

**Figure 7:**
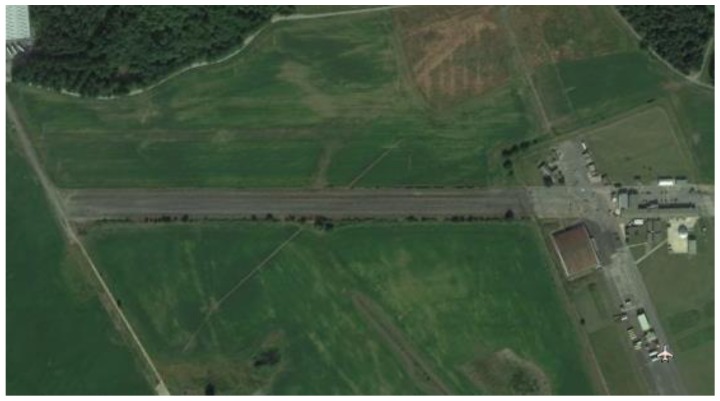
An old airplane runway in Worcestershire, United Kingdom.

The test runs were conducted on an old airplane runway in Worcestershire, United Kingdom. Figure 7 shows a straight and flat asphalt surface of 0.6 miles length.

## 5. Experimental Results and Discussion

This section presents the performance of the two algorithms tested using experimental data. Two sets of experiments were performed. The first set consists of driving on flat road at different speeds and the second set is comprised of driving the test vehicle on different types of surfaces.

### 5.1. Test 1: Accuracy of Algorithms at Different Speeds

**Figure 8:**
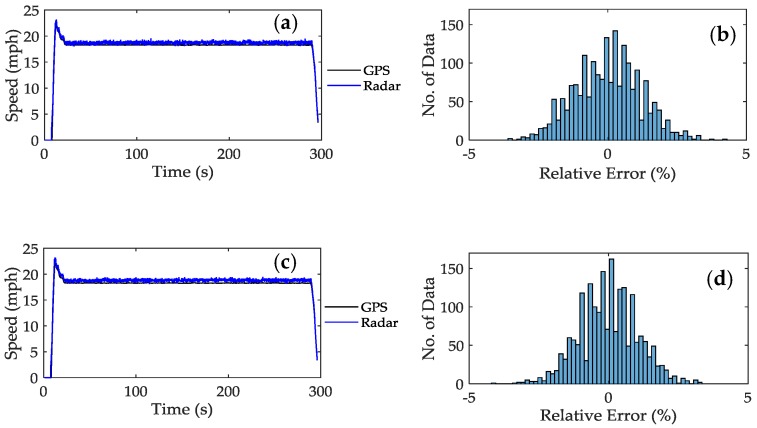
The actual radar and GPS speed at approximately *v* = 20 mph (**a**) CMA and (**b**) XCA. The distribution of speed error at *v* = 20 mph (**c**) CMA and (**d**) XCA.

We collected time-series Doppler data for speed of *v* = 10, 20, 30, 40 and 70 mph from the 0.6-mile straight road. Speed estimated from each sensor is computed at every 100 ms and the sum of the longitudinal speed is compared to the speed collected using the GPS receiver. Figure 8a,c show the qualitative plot of the speed estimated using the CMA and XCA respectively. In both graphs, it can be observed that the estimated speed closely tracks the GPS reference speed during the rapid acceleration, constant speed and deceleration. The performance of the algorithms is measured in terms of relative error and it is given as

(16) $$\mathrm{Relative}\;\mathrm{error}\;\left(\%\right)=\left(\frac{v_{radar}-v_{GPS}}{v_{GPS}}\right)100$$

where *v_radar_* and *v_GPS_* are the speed measured using radar and GPS respectively. Figure 8b,d show the distribution of speed error estimated using the CMA and XCA respectively. Both algorithms produce estimates that closely follow a normal distribution. Similar results were also observed in the rest of the measured speeds; hence, the plots are not shown here. Instead we tabulated the estimation performance in terms of average relative error, maximum relative error and the percentage of speed values falling within ±1% of the true reference speed.

From Table 2, we observe that both methods show remarkable accuracy, with more than 50% of the speed estimates falling in the 1% error band with an exception for CMA at *v* = 10 mph. Overall, the XCA produces more accurate estimates than the CMA. On average, 72% of XCA estimates fall within the 1% gap compared to 60.5% for CMA. The XCA also produces smaller maximum error compared to the CMA.

### 5.2. Test 2: Accuracy of Algorithms on Different Terrain

We collected Doppler data from four different terrains around the vicinity of the 0.6-mile road. Table 3, below, briefly describes the terrains.

The test vehicle was driven with appropriate speed between 0 and 10 mph and in a linear path during the data collection. The data is processed in similar way as in Test 1. Table 4 below summaries the estimation accuracy of both algorithms.

**Figure 9:**
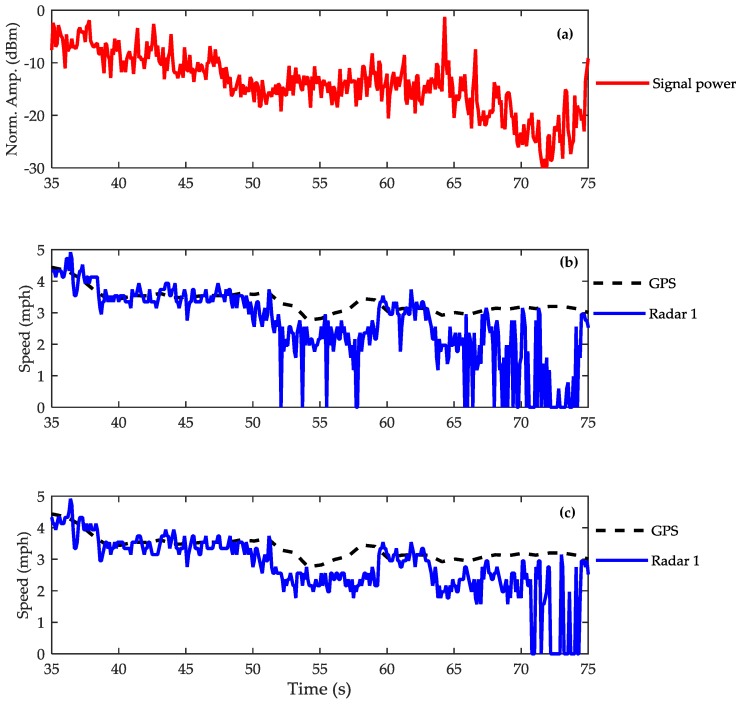
The return power and the estimated speed by algorithms (**a**) Radar 1 return power; (**b**) CMA estimated speed; (**c**) XCA estimated speed.

We observed that the estimation error for both algorithms is higher than estimates on the flat surface. Unlike in Table 2, where the XCA performs better than the CMA, the accuracy of the two algorithms in this test is comparable in average and maximum errors. An exception can be seen on the water surface test, where the CMA produced considerably larger error than the XCA. To understand more on this issue, a more detailed examination should be performed, looking at the return echo from water surface. Figure 9a shows the radar return power measured from a sensor travelling from a wet-earth surface to a long pothole of water. The length of the water surface is between the 50th and 75th s. The degradation in return power is approximately 10 dB lower on the water surface. The smallest return power was recorded between the 70th and 75th s, where we observed the water to be at its calmest. Figure 9b,c show the speed estimation results obtained using the CMA and the XCA respectively. Comparing the plots in Figure 9b between the time of the 50th and 60th s with the corresponding ones in Figure 9c, we can observe that the estimated speed using CMA exhibits unreliable estimation where the speed drops to *v* = 0 mph four times. This is because we intentionally set the algorithms to return zero speed to indicate a failure in the Doppler shift computation. Evidently from Figure 9c, we can say that the XCA outperform the CMA for low SNR echoes. We observed both algorithms estimated lower speed than the reference speed of about 0.4 ms^−1^. This can be seen clearly in the period from the 52nd to 60th and from the 63rd to 66th s. The experiment also shows that when water is calm, neither type of algorithm works in this condition.

### 5.3. Discussion

From Table 2, the XCA presents to be the optimal algorithm of the two. It produces a more accurate estimation in the ±1% error band compared to the CMA. This is due to the characteristics of Doppler spectra collected from the flat road surface where the shape of the spectra closely follows the shape of the antenna pattern. In addition, the cross-correlation is known to provide the best results when the amplitude variation in the Doppler spectrum has the characteristics of white Gaussian noise. Another important fact from Table 2 is that the number of accurate estimates increases with the vehicle speed. This suggests that as the Doppler spectrum broadens and that, provided a sufficient SNR, it is much easier for the algorithms to differentiate the shape of the echo from random noises in spectrum. A Doppler echo at low speed has a narrow bandwidth, and depending on number of FFT used, the echo may be presented by a small number of frequency bins. Thus, the amplitude variation within the narrow bandwidth alone can reduce the accuracy of estimation. The problem can be further aggravated with the existence of 1/f noise near the echo. One way to improve accuracy in the low-speed estimation is by using a lower sampling rate, which sufficiently covers the Nyquist bandwidth of low-speed Doppler spectra.

The increase in errors in the off-road test is due to several factors. The vibration, pitch and roll of the test vehicle during the experiment, changes the look angle, *θ_i_* of the sensors. The Janus sensor arrangement functions to reduce the error due to the change in *θ_i_*, but it cannot eliminate the error. Secondly, the echo from off-road may slightly change due to the surface characteristics which reduces the effectiveness of the cross-correlation method. The backscattering from the ground surface is a superposition of many weak signals scattered by small irregularities of the ground surface. These irregularities are random, and therefore the backscattering is different from one part to another. In addition, the effective illuminated area of the radar is dependent on radar cross section (RCS) of the ground surface and thus dependent on the size of the illumination surface and the backscattering coefficient of the ground. Particularly wet asphalt is known to have lower backscattering coefficient compared to dry asphalt surface [28].

Limiting SNR is one of the most important aspects in measuring the performance of an algorithm. It is necessary to know the minimum level of SNR at which the algorithm can work accurately. Water surface is known to weaken the return echo of a radar [29]. Here, we tested the algorithms with the Doppler data collected from a water surface. The result shows that the XCA has advantages in this condition. This is because the cross-correlation estimates the Doppler shift using spectrum shape. On the other hand, CMA uses frequency amplitude to discriminate between noise and useful signal.

Finally, in addition to the results obtained, the accuracy and the cost-effectiveness of the overall solutions, one of the objectives is to design algorithms that facilitate a simple implementation of algorithms in actual SoG systems, rather than the development of a bespoke radar system. These algorithms can take the advantages of many efficient FFT-processors that are already available on the market, such as [30].

## 6. Conclusions

This work has brought forward two algorithms for estimating SoG for cars. The first algorithm searches the center of mass of the spectrum and uses the center as Doppler shift. The second method searches for the best match shape and uses the highest correlation index as Doppler shift. Both methods had unique pre-processing to evade and reduce the effects of noisy spectrum in the determination of Doppler shift. Both algorithms have their own relative merits and drawbacks, so to evaluate them for practical automotive applications a dedicated experimental campaign was carried out on different road conditions.

Both algorithms produced sufficiently accurate speed estimation results and are of low computational cost, but the performance of one algorithm was found to be better for off-road conditions. However, both algorithms were found suitable overall for the practical implementation of SoG radar systems. Moreover, the radar system brought forward is in line for future trends in the automotive industry, which include the addition of more radar sensors on cars including at their corners. This means that in a practical environment, the proposed solution could re-use radar data from these sensors, and therefore it would not only be of sufficient accuracy and efficiency, but also of substantially low cost as a software-only solution.

As a future work, research could be undertaken to identify alternative approaches for radar SoG estimation. One of these approaches could be the analysis of the phase history of the signal as the vehicle traverses some distance, in a Synthetic Aperture Radar (SAR)-like approach, rather than the magnitude and shape of the instantaneous Doppler spectrum. At a high level, it is generally known that phase is more sensitive than amplitude, so such a technique could improve SoG accuracy even further. However, the development of such a technique would be a new topic for study whose accuracy and practicality for SoG tasks should be assessed at the proof-of-concept level. 

## Figures and Tables

**Table 1:** The average time for algorithms to complete one successful estimate of mean frequency.

No	FFT Length	Duration of Samples (ms)	CMA (ms)	XCA (ms)
**1**	3125	100	0.01	0.6
**2**	6250	250	0.01	0.8
**3**	12,500	500	0.03	1
**4**	25,000	1000	0.04	2

**Table 2:** The performance of the algorithm at speed 10 to 70 mph (**a**) Average relative error (**b**) Maximum relative error (**c**) The percentage of speed values falling within ±1% of the true reference speed.

**Speed**	**10 mph**	**20 mph**	**30 mph**	**40 mph**	**70 mph**
XCA	1.2%	1.0%	0.7%	0.6%	0.5%
CMA	1.4%	1.0%	0.9%	0.8%	0.8%
(a)					
**Speed**	**10 mph**	**20 mph**	**30 mph**	**40 mph**	**70 mph**
XCA	5.5%	4.1%	3.0%	2.3%	1.9%
CMA	6.7%	4.8%	4.7%	6.2%	3.1%
(b)					
**Speed**	**10 mph**	**20 mph**	**30 mph**	**40 mph**	**70 mph**
XCA	50.6%	57.9%	77.0%	85.1%	89.0%
CMA	42.2%	55.0%	63.4%	70.8%	71.4%
(c)					

**Table 3:** The definitions of surfaces in Test 2.

Surface	Visual Description
Grass	Grass-covered surface with grass height approximately between 1 and 7 cm. The road surface is approximately even
Bumpy	Aged asphalt road with many potholes and an uneven surface. The depth of the potholes is approximately between 5 and 7 cm
Wet Dirt	Dirt road consisting largely of dirt and small gravel. The surface is uneven and has potholes with depth approximately between 3 and 5 cm and filled with rain water
Water	A 5-m width dirt road completely covered with murky water of depth approximately 10 cm

**Table 4:** Algorithm performance (**a**) Averaged relative error (**b**) Maximum relative error (**c**) The percentage of speed values falling within ±5% of the true reference speed.

	**Grass**	**Bumpy**	**Wet Dirt**	**Water**
XCA	5.0%	6.4%	6.7%	8.0%
CMA	5.0%	6.2%	8.2%	29.2%
(a)				
	**Grass**	**Bumpy**	**Wet Dirt**	**Water**
XCA	18.7%	23.1%	34.0%	35.1%
CMA	18.4%	21.5%	40.3%	122.0%
(b)				
	**Grass**	**Bumpy**	**Wet Dirt**	**Water**
XCA	57.1%	47.0%	46.0%	34.0%
CMA	59.6%	49.4%	41.4%	19.0%
(c)				
